# Fluorescein-guided resection of newly diagnosed high-grade glioma: Impact on extent of resection and outcome

**DOI:** 10.1016/j.bas.2022.101690

**Published:** 2022-11-09

**Authors:** Karl-Michael Schebesch, Julius Höhne, Katharina Rosengarth, Ekaterina Noeva, Nils Ole Schmidt, Martin Proescholdt

**Affiliations:** a Department of Neurosurgery, University Medical Center Regensburg, Regensburg, Germany; b Wilhelm-Sander Neuro-Oncology Unit, University Medical Center Regensburg, Regensburg, Germany; c Neuroradiology Branch, Department of Radiology, University Medical Center Regensburg, Regensburg, Germany

**Keywords:** Fluorescein sodium, Fluorescence-guided surgery, High-grade glioma, Neuro-oncologic surgery, Extent of resection, Malignant brain tumors, Neuro-oncology

## Abstract

**Introduction:**

Maximal resection of high-grade glioma (HGG) improves progression–free survival (PFS) and overall survival (OS). Fluorescein sodium (FL) in combination with the YELLOW 560 ​nm filter (Carl Zeiss Meditec, Germany) is a safe and feasible method of visualizing residual tumor tissue during brain tumor resection.

**Research question:**

We hypothesized that use of FL positively influenced the volumetric extent of resection (EOR), PFS, and OS in patients undergoing resection of a newly diagnosed HGG.

**Materials and method:**

Using a prospective HGG registry, we identified 347 patients (median age 62.4 years; 141 women) with preoperative high-quality magnetic resonance images for volumetric analysis. Resection was performed under white light in n ​= ​151 (43.5%, white-light group) and under FL-guidance in n ​= ​196 (56.5%, FL group). Sex, age, presurgical Karnofsky Performance Index (KPI), O6-Methylguanin-DNA-Methyltransferase-Gene (MGMT) status, and adjuvant treatment modalities were well balanced between the groups. Volumetric analysis was performed by quantifying pre- and postoperative tumor volume based on gadolinium-enhanced T1 sequences in a blinded fashion.

**Results:**

In the FL group, postoperative tumor volume was significantly smaller (p ​= ​0.003); accordingly, quantitative EOR was significantly larger (p ​= ​0.003). Significantly more complete resections were achieved in the FL group than in the white-light group (p ​= ​0.003). The FL group showed significantly longer PFS (p ​= ​0.020) and OS (p ​= ​0.015, log rank testing). Multivariate Cox regression modelling showed age, presurgical KPI, MGMT status, and FL-guided resection to be independent prognostic factors for survival.

**Discussion and conclusion:**

Compared to white-light resection, FL-guided resection of newly diagnosed HGG significantly improved EOR and prolonged OS.

## Highlights

• Post-hoc analysis of a prospective database (GlioOutcome) of 347 patients with high grade gliomas.
• 196 patients assigned to fluorescence-guidance group and 151 patients assigned to the white light illumination group.
• Statistical analysis revealed an improved rate of complete resections in the fluorescence group (p=0.003).
• In the fluorescence group we found a prolonged progression-free survival (p=0.20).
• Prolonged overall survival for patients of the fluorescence-guidance group was achieved (p=0.015).
• We ​conclude that fluorescence-guided resection is superior to white light resection.

## 1 Introduction

The fluorescent dye fluorescein sodium (FL) has been used in neurosurgical oncology worldwide for nearly a decade, mainly in combination with a dedicated light filter (e.g. YELLOW 560 ​nm filter, Carl Zeiss Meditec, Oberkochen, Germany) (Acerbi et al., 2021). Recent reviews and meta-analyses have yielded a significant increase in the quality of resection of high-grade glioma (HGG) and brain metastases. The same applies to various tumors of different etiologies that manifest in the human central nervous system by disturbing the intrinsic blood brain barrier (BBB) (Mazurek et al., 2020; Cavallo et al., 2018; Schebesch et al., 2016; Ahrens et al., 2022). Some authors have even concluded that the still off-label FL dye is non-inferior to the established and approved fluorescence marker 5-aminolevulinic acid (5-ALA) with regard to the extent of resection (EOR), progression-free survival (PFS), and overall survival (OS), although these conclusions are based on different levels of evidence (Zeppa et al., 2022; Naik et al., 2022). In a retrospective monocentric analysis, Hansen et al. found that patients with HGG had significantly increased PFS after FL-guided resection than after 5-ALA- guided resection (Hansen et al., 2019).

However, data on the clinical benefits of FL-guided resection in patients with HGG are sparse. The publications available so far only comprise a limited number of patients or have substantial limitations due to selection bias or insufficient quality of the data (Naik et al., 2022).

In 2013, our neurosurgical department was one of the first to implement FL-guided neuro-oncologic surgery according to an institutional standard (Schebesch et al., 2013). By now, we have extensive experience obtained from conducting a large number of surgical interventions as well as two prospective multicenter trials FLUOGLIO (Acerbi et al., 2018) and INVIVO (unpublished data).

### 1.1 Research question

For this reason, we are able to present a large retrospective high-quality group comparison of volumetrically evaluated EOR, PFS, and OS between conventional white-light surgery and FL-guided surgery in patients with HGG. The presented data analysis will significantly increase scientific knowledge about the potential benefit of FL-guided surgery.

## 2 Materials and method

Screening of our prospective HGG-bioregistry (‘*GlioOutcome’,* started in 2013) yielded 347 patients (141 women, mean age 62.4 years) with newly diagnosed HGG. Inclusion criteria were contrast-enhancing mass suggestive of HGG in preoperative T1-weighted magnetic resonance imaging (MRI), potential resectability of the lesion, final histopathological diagnosis of glioblastoma WHO grade IV or isocitrate-dehydrogenase-1 (IDH-1) mutation astrocytoma WHO grade IV, early postoperative contrast-enhanced MRI for volumetric analysis, complete datasets of adjuvant treatment, as well as radiographic and clinical follow-up data as indicated by the interdisciplinary tumor board. We excluded recurrent HGG, low grade glioma (WHO I, II), and all patients who were lost to follow-up.

Overall, 151 patients had been treated surgically under white light (43.5%), and 196 patients had received FL-guided resection (56.5%). The indication for white-light surgery were contraindication for the use of FL (use of beta-blockers, renal or hepatic dysfunction, or known intolerance of FL), and absence of informed consent for the off-label use of FL. We detected no statistically significant differences between the two groups concerning patient sex, age, pre-operative neurological performance according to the Karnofsky Score (KPS) or the O6-Methylguanin-DNA-Methyltransferase-Gene Status (*MGMT*), and adjuvant treatment. The baseline data of the two populations are shown in Table 1.

**Table 1:** Baseline data of the population, distributed in the white-light group and the FL group (f means female, m means male, KPS means Karnofsky Performance Score).

Parameter	White-light group	FL group	p
N ​=	151 (43.5%)	196 (56.5%)	
Sex (f/m)	64/87(42.4%/57.6%)	77/119 (39.3%/60.7%)	0.560
Age (median)	61.9 years (range: 32.1–87.8)	62.6 years (range: 24.2–86.6)	0.202
Preoperative KPS (median)	80 (range: 50–100)	80 (range: 50–100)	0.696
MGMT			0.125
Methylated	62 (41.1%)	96 (48.9%)	
Unmethylated	81 (51.6%)	96 (48.9%)	
Unknown	8 (5.3%)	4 (2.2%)	
Postsurgical treatment			0.782
Concomitant radio-chemotherapy	110 (72.8%)	152 (77.5%)	
Radiation only	15 (9.9%)	15 (7.7%)	
Chemo only	17 (11.3%)	15 (7.7%)	
No treatment	9 (6.0%)	14 (7.1%)	

In both groups, patients routinely received intraoperative sonography and neuro-navigation. Patients in the FL group received 5 ​mg fluorescein sodium 10% (ALCON, Germany) per kilogram bodyweight during the induction of anesthesia via the central venous line approximately 30 ​min prior to skin incision. The YELLOW 560 ​nm filter (Carl Zeiss Meditec, Oberkochen, Germany) was applied for visualizing fluorescence during resection. The institutional standard of FL application has been published previously (Schebesch et al., 2016). A common strategy to avoid the unintended removal of false-positive fluorescence due to manually-induced additional BBB disruption is to place a piece of cotton onto the non-fluorescent surgical field immediately after the fluorescent tumor tissue has been removed; this way, re-resection can be prevented.

A blinded neuroradiologist performed quantitative volumetric analysis of the preoperative and postoperative T1-weighted, contrast-enhanced sequences. The images were transferred to Brainlab iPlan cranial (Brain Lab, Munich, Germany) software for further assessment.

Procedure-related morbidity were defined as occurrence of infection of the surgical area, cerebro-spinal fluid (CSF) fistula, cranial hemorrhages, pulmonary embolism, cardiac complication, all within 30 days postoperatively.

The study was approved by the institutional review board (Z-2015-0478-9, Ethics Committee of the University of Regensburg).

### 2.1 Statistical analysis

Descriptive statistical analysis was performed by reporting continuous variables as median and ranges and categorical values as counts and percentages. PFS and OS data were determined by applying the Kaplan-Meier estimator. Factors associated with prolonged survival were evaluated as univariate analysis by calculating log rank tests. Multivariate analysis of independent prognostic factors was performed using Cox proportional hazards modelling. Results with a p-value <0.05 were considered statistically significant. Analyses were obtained using Stata/IC (version 16.1, Stata corp. College Station, USA).

## 3 Results

**Fig. 1:**
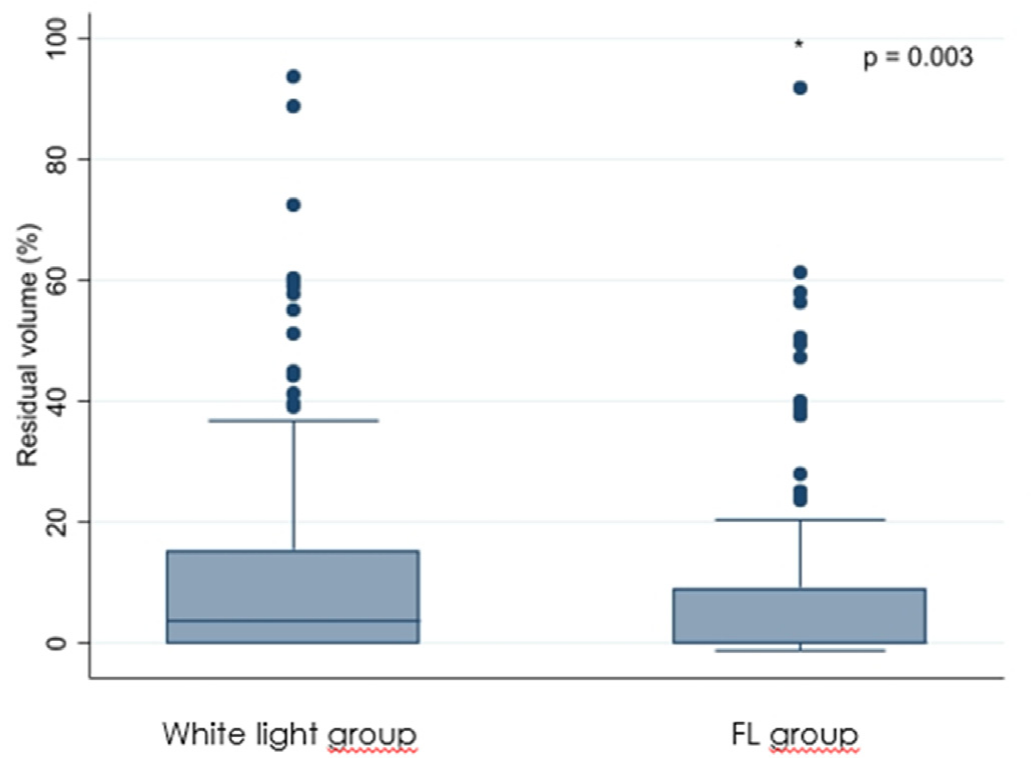
Graphical plot showing a significant difference in residual tumor volume between the two groups.

**Fig. 2:**
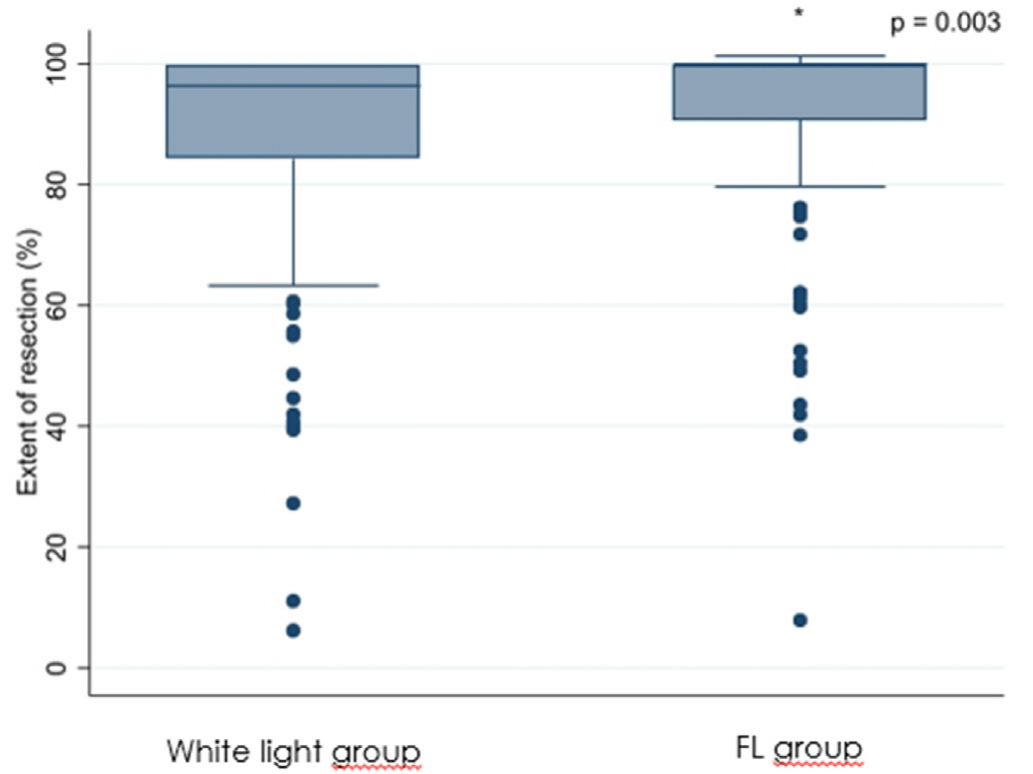
Graphical plot showing a significant difference in the extent of resection between the two groups.

There was no statistically significant difference in volumetrically assessed preoperative tumor volume between the white-light group and the FL group (p ​= ​0.065) but in volumetrically assessed postoperative tumor volume (p ​= ​0.003), EOR (p ​= ​0.003), PFS (p ​= ​0.020), and OS (p ​= ​0.015), see Table 2 and Fig. 1, Fig. 2, Fig. 3b, Fig. 3a.

**Table 2:** Results; differences between the white-light group and the FL group.

Parameter	White-light group	FL group	p
N ​=	151 (43.5)	196 (56.5)	
Preoperative tumor volume (median; ml)	25.6 (range: 2.2–117.3)	29.5 (range: 2.4–109.8)	0.065
Residual volume (median; ml)	3.6 (range: 0.0–93.8)	0.0 (range: 0.0–92.0)	0.003
Extent of resection (median; %)	96.4 (range: 44.8–100.0)	100.0 (range: 61.6–100.0)	0.003
Progression-free Survival (median, months)	6.94 (range: 1.5–63.6)	8.12 (range: 1.4–70.2)	0.020
Overall survival (median; months)	15.5 (range: 1.9–118.9)	16.7 (range: 2.7–74.6)	0.015

**Fig. 3a:**
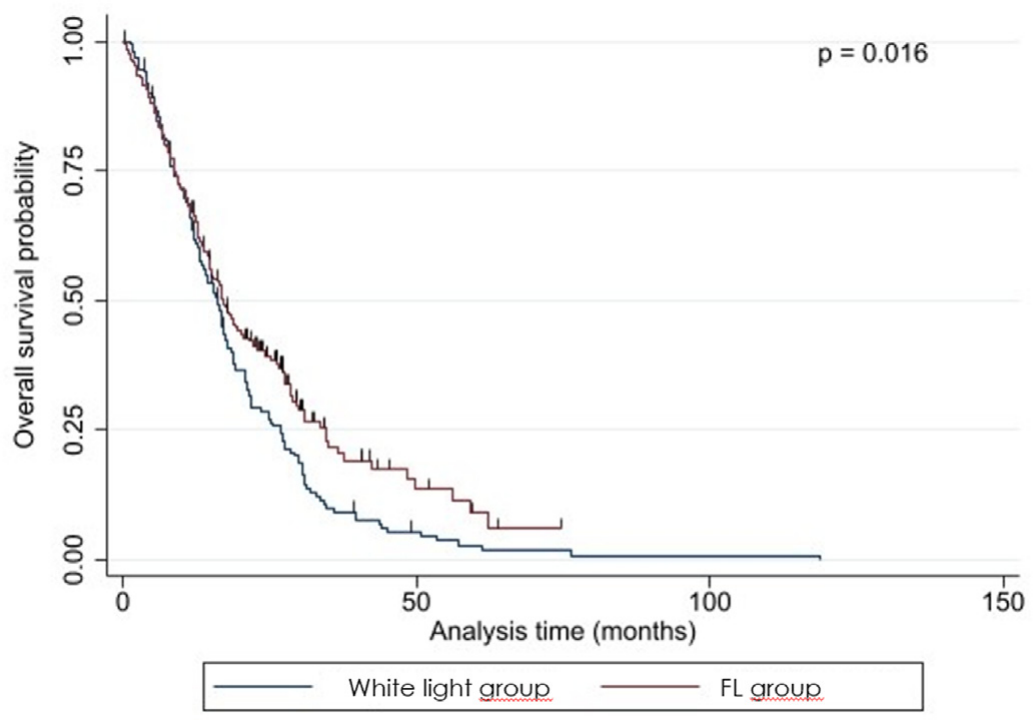
Kaplan-Meier graph for overall survival showing a significant difference between the two groups.

**Fig. 3b:**
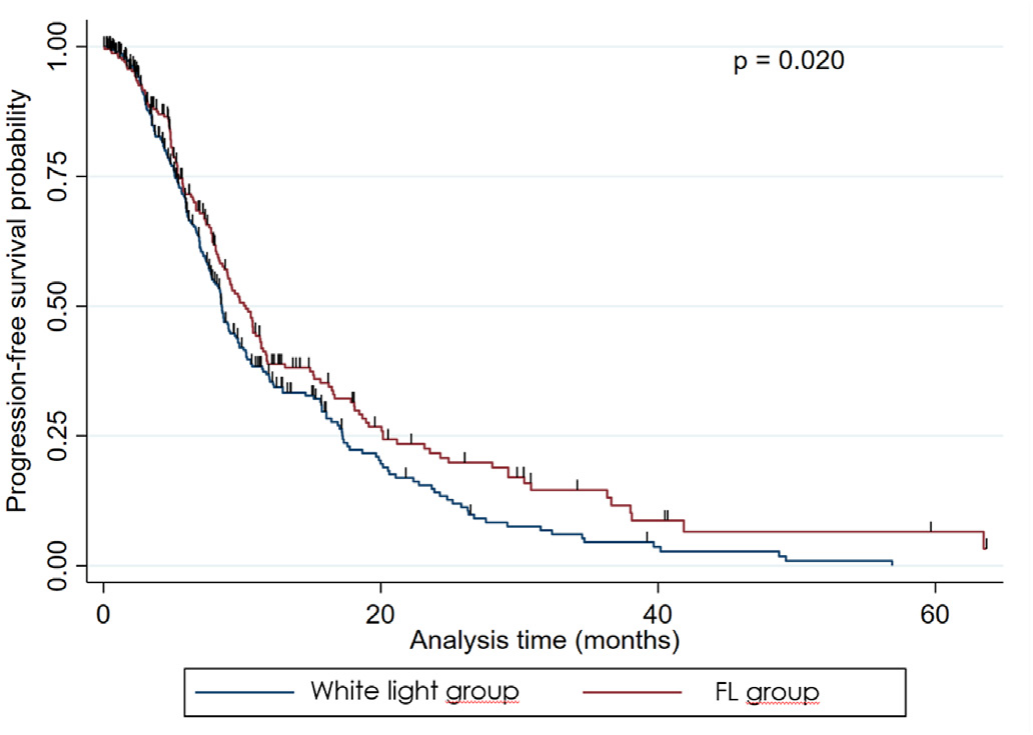
Kaplan-Meier graph for progression-free survival showing a significant difference between the two groups.

Multivariate Cox regression model for OS showed age (p ​= ​0.001), preoperative KPI (p ​= ​0.012), MGMT-promoter status (p ​= ​0.001), and FL-guided resection (p ​= ​0.006) to be significantly predictive factors, see Table 3.

**Table 3:** Results; Multivariate Cox regression model for overall survival.

Parameter	Hazard Ratio	95% CI		p ​=
Age	1.023	1.012	1.035	0.001
KPI presurgical	0.986	0.976	0.997	0.012
MGMT status	1.646	1.313	2.062	0.001
FY resection	0.719	0.569	0.908	0.006

In the FL group, 91 (46.4%) tumors were located next to an eloquent area, whereas 59 tumors (39.1%) in the white-light group had developed next to an eloquent area. This difference was not significant (p ​= ​0.077).

Each surgery was performed by or under the supervision of an experienced tumor surgeon. Surgical mortality (p ​= ​0.545) and morbidity (p ​= ​0.314) did not significantly differ between the groups. In particular, we did not find any differences in the frequency and severity of transient or permanent neurological deterioration (p ​= ​0.753). We did not find any adverse events or anaphylactic reactions due to the administration of FL. The majority of patients showed extensive, but transient, yellowish staining of the urine caused by the exclusively renal excretion of FL within 24 ​h.

For illustrative purposes, we present the intraoperative visualization of the surgical field prior to and during the resection of HGGs. Surgeons may easily distinguish between the fluorescent and non-fluorescent areas under the filtered light (Fig. 4a, Fig. 4ba–d).

**Fig. 4a:**
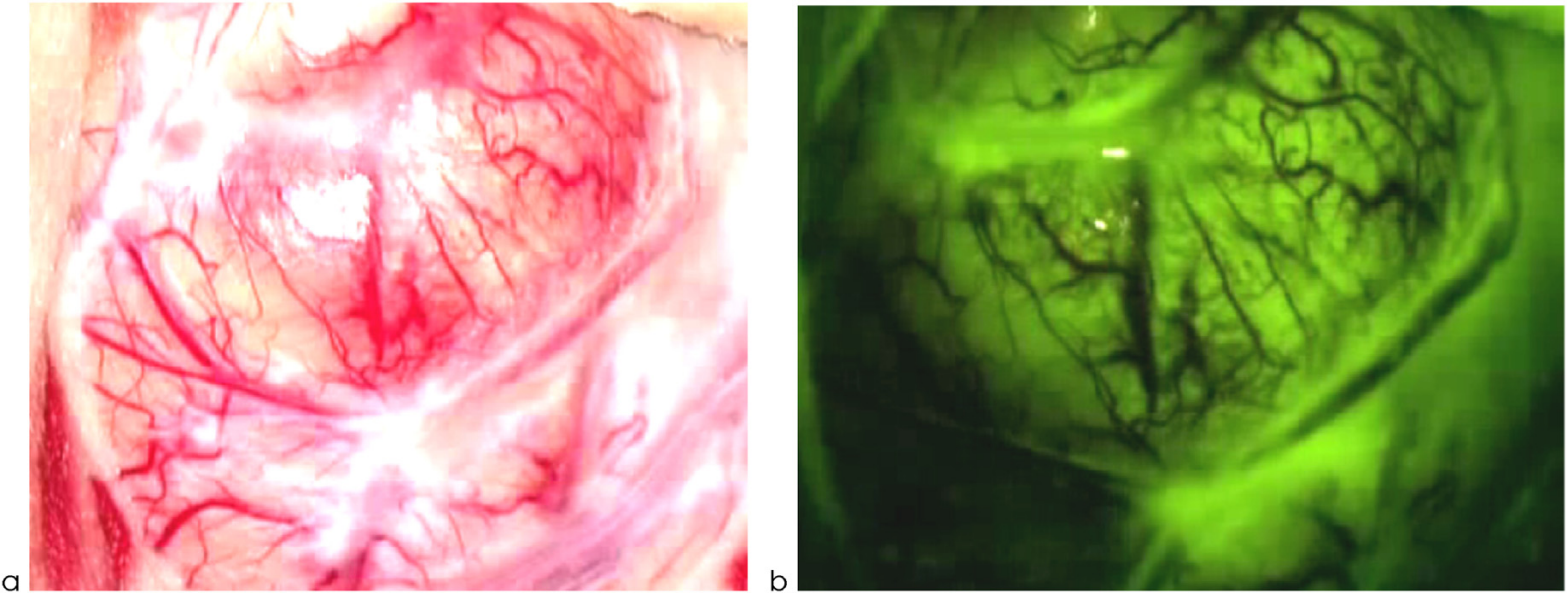
Intraoperative pictures of HGGs prior to resection under white light (a) and under filtered light (b).

**Fig. 4b:**
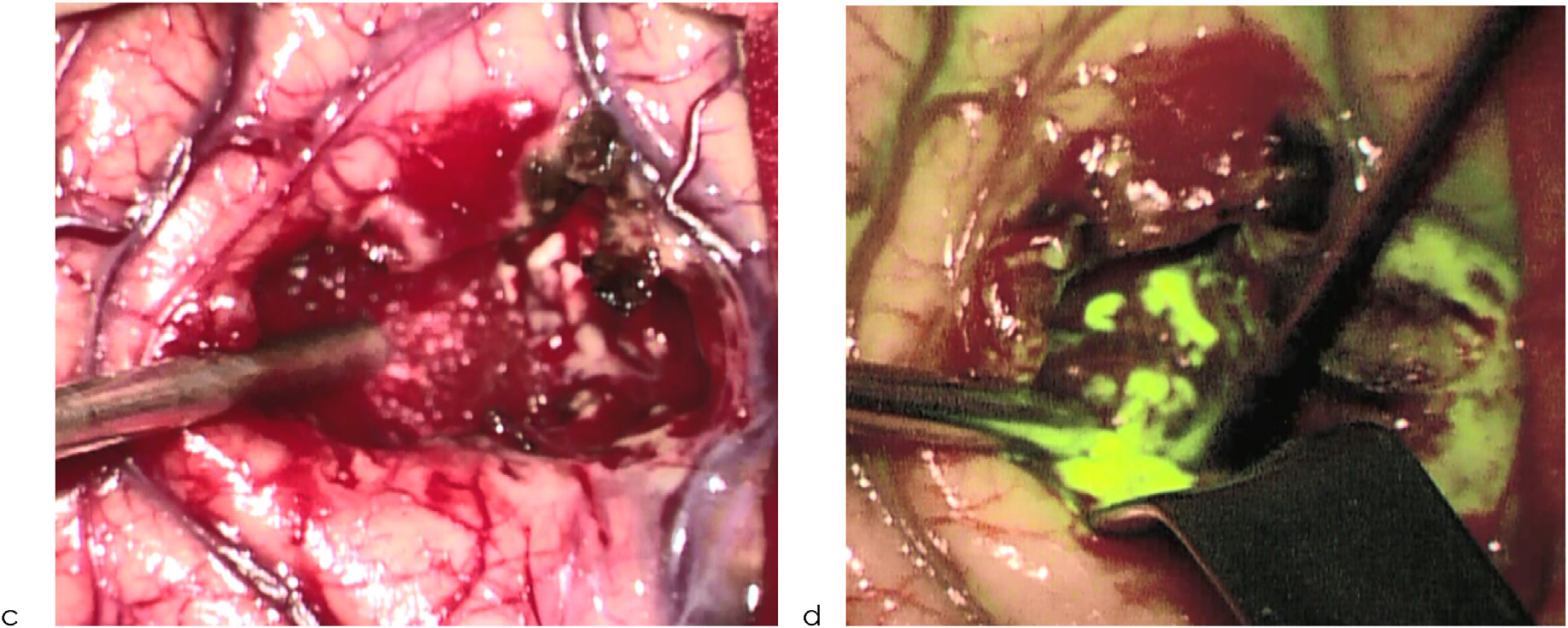
Intraoperative pictures of HGGs during resection under white light (a) and under filtered light (b).

## 4 Discussion and conclusion

According to current knowledge, complete resection of HGG is the most beneficial basis for any adjuvant treatment and prolongs PFS and OS (Sanai et al., 2011; Lacroix et al., 2001; Weller et al., 2017). The radiographic parameter for assessing the EOR is still the gadolinium-enhanced T1-weighted MRI sequence generated within 48 ​h after surgery (Weller et al., 2017). According to comparable molecular weight and similar biological behavior in depicting the disrupted BBB (Xiang et al., 2018), the intraoperative appearance of FL accurately resembles gadolinium enhancement in MRI (Neira et al., 2017). Some authors, however, have suggested to achieve supramarginal resection in HGG, but no study has yet effectively proven the practicability and positive survival effect of resections beyond the area of contrast enhancement. Furthermore, the diagnostic tools for measuring the EOR exceeding visible BBB disruption are still controversially discussed (Guerrini et al., 2022).

In the current literature, FL is being increasingly investigated beyond the simple feature as a fluorescent dye. Some interesting articles outlined its usefulness in augmented reality high-definition fiber tractography (Luzzi et al., 2021) and as a diagnostic marker for intraoperative confocal laser-endomicroscopy (Höhne et al., 2021). Nevertheless, the safety and efficacy of FL for easy-to-use fluorescence-guided resection remains its most important attribute.

As shown by pathological gadolinium uptake, FL enhances the quality of resection of any cerebral or spinal lesion with consecutive BBB disturbance (Falco et al., 2019), see Fig. 4b, Fig. 4aa–d. This effect was also shown in the prospective multicenter FLUOGLIO trial in 2017 (Acerbi et al., 2018); however, that uncontrolled study only had a limited number of patients (n ​= ​57), and statistical restrictions did not allow establishing any dedicated survival benefits.

With our analysis, we sought to undermine the eligibility of FL in combination with a dedicated light filter. On the one hand, we present a large number of patients with HGG who received surgery under FL-guidance and a comparable control group with volumetrically assessed pre- and postoperative MRI data in a blinded fashion. On the other hand, we were able to determine progression-free and overall survival due to the high-quality data obtained from our prospective database. The present study yielded increased EOR, PFS, and OS after FL-guided resection. All demographic parameters were well balanced between the two groups and in addition, the adjuvant treatment regimen was identical. This indicates that the PFS and OS benefit exclusively resulted from the improved EOR according to fluorescence-guidance.

Our study showed a slight disbalance in the MGMT promoter methylation status, which may possibly influence survival results and therefore interfere with the comparison between white-light and FL-guided resection. In the multivariate analysis, however, we included both the MGMT promoter methylation status and the type of resection into our Cox regression model. These results indicate that FL-guided resection is a positive prognostic factor for affecting survival independently of the MGMT promoter methylation status. In addition, our survival interaction analysis for the factors MGMT promoter methylation status and type of resection did not show any significant interference between these two parameters (p ​= ​0.628).

Few previous studies comparing the quality of resection under FL and simple white light have been conducted so far, and each of them has significant limitations. Some studies had a very limited number of patients (Catapano et al., 2017; Chen et al., 2012), some studies did not include the use of a dedicated light filter and thus required high doses of FL (Chen et al., 2012; Koc et al., 2008; Shinoda et al., 2003), and, in some studies, rather old data (>20 years) had to be consulted as control (Katsevman et al., 2019). Furthermore, strict volumetric analyses of preoperative and postoperative MRI data are lacking completely, even in the most recent publication by Hong et al. with 82 patients that yielded a gross-total resection (GTR) rate of 85.7% in the FL group versus 62.5% in the white-light group (Hong et al., 2019).

Moreover, reliable data about the influence of FL-guided resection on PFS and OS are rare. Two studies did not find any significant differences between conventional and FL-guided surgical interventions (Koc et al., 2008; Shinoda et al., 2003), and only one small study by Chen et al. (n ​= ​26) documented a statistically significant increase in PFS after FL-guided resection (Chen et al., 2012).

In their comprehensive review, Ahrens et al. thus demanded further studies to contribute to the sparse data on the potential benefit of using FL in HGG surgery because the level of evidence regarding FL-guided surgery in comparison to surgery under white light was found to be level III and below (Ahrens et al., 2022).

Consequently, we believe that our study supports the positive effect of FL-guided surgery on radiographic outcome and survival. The design of the present study, however, is monocentric post-hoc analysis—albeit the data were extracted from a prospective registry—so that the study presented here has the limitations of any retrospective analysis. In our neuro-oncologic center, however, the indication for and the protocol of FL administration has been established in a standardized manner since 2014 ​at the latest. Additionally, the radiographic and neuro-oncologic conditions have not been significantly modified so that patients of both groups were adequately treated according to international conventions and contemporary recommendations by the interdisciplinary tumor board (Haj et al., 2017). With this precondition and a homogeneous distribution of molecular specifications such as the MGMT-promoter status, we postulate that the data quality of our presented series is valid.

Notwithstanding, a reliable conclusion whether FL-guidance is equivalent to 5-ALA guidance cannot be drawn directly. This question was addressed in the 2022 network meta-analysis by Naik et al., who showed that FL-guided and 5-ALA-guided techniques have similar beneficial effects on EOR and OS (Naik et al., 2022).

Obviously, prospective data on a larger scale are required. Yet, after the 2021 publication of a pooled analysis including >330 patients with successful FL-guided surgery for HGG (Smith et al., 2021), we feel that treating randomly assigned patients with HGG under white light is more than problematic for ethical reasons.

To the best of our knowledge, the present study is the largest comparison between FL-guidance and white-light guidance in HGG surgery worldwide. Our results strongly support the notion that FL-guided surgery is a safe and feasible method that significantly increases the quality of resection because it prolonged PFS and OS in our patients without any relevant increase in perioperative or postoperative morbidity and mortality.

## Funding

None.

## Conflict of interest

K.M.S. and J.H. received financial support, travel fees, and honoraria from Carl Zeiss Meditec, Germany.

## Declaration of competing interest

The authors have no competing interests.
